# Multidistrict invasive *Candida glabrata* infection successfully treated with echinocandin and liposomal amphotericin B combination therapy: a case report and therapeutic perspective

**DOI:** 10.3389/fmed.2026.1796594

**Published:** 2026-04-16

**Authors:** Massimo Caracciolo, Francesco D’Aleo, Maria Francesca Stagno, Carmelo Papola, Simona Pellicano, Giovanna Maria Nicolò, Sarah Antonella Caracciolo, Antonino Ripepi, Nadia Pellicano, Clara Scopelliti, Luigi Principe, Stefano La Scala

**Affiliations:** 1 UOSD Post-Operative Intensive Care Unit, Great Metropolitan Hospital “Bianchi Melacrino Morelli”, Reggio Calabria, Italy; 2 Clinical Microbiology and Virology Unit, Great Metropolitan Hospital “Bianchi Melacrino Morelli”, Reggio Calabria, Italy; 3 UOC Perioperative and General Anaesthesia and Intensive Care, Senese University Hospital, Siena, Italy

**Keywords:** antifungal resistance, *Candida glabrata*, case report, combination therapy, echinocandins, invasive candidiasis, liposomal amphotericin B

## Abstract

**Introduction:**

*Candida glabrata* is the second leading cause of invasive candidiasis worldwide and represents a major clinical challenge due to its intrinsic antifungal resistance, stress tolerance, and ability to persist in hostile host environments. We report a complex case of multidistrict *C. glabrata* infection in a 39-year-old immunocompetent male who presented with septic shock secondary to gastric perforation and thoracic contamination. Despite guideline-recommended echinocandin therapy, persistent fungal growth was detected in multiple anatomical compartments, including bloodstream, pleural fluid, surgical wound, and bronchoalveolar lavage samples.

**Methods:**

Serial cultures and antifungal susceptibility testing were performed using MALDI-TOF MS and interpreted according to CLSI criteria. Radiological monitoring, surgical source control, and pharmacokinetic considerations guided therapeutic decisions.

**Results:**

*C. glabrata* isolates showed reduced azole susceptibility but remained susceptible to echinocandins and amphotericin B. Persistent fungal growth between postoperative day (POD) 8 and POD 16 prompted escalation from caspofungin to micafungin and subsequently to combination therapy with liposomal amphotericin B. Rapid clinical improvement followed initiation of dual therapy, with microbiological clearance by POD 20 and complete recovery by POD 30.

**Conclusion:**

This case highlights the challenges of treating multidistrict *Candida glabrata* infections and underscores the potential role of echinocandin–liposomal amphotericin B combination therapy when pharmacokinetic barriers may limit the effectiveness of antifungal monotherapy.

## 1 Introduction

*Candida glabrata* is a ubiquitous opportunistic yeast that has emerged as the second most common cause of invasive candidiasis worldwide, surpassed only by *Candida albicans* (1, 2). It is responsible for a broad clinical spectrum of disease, ranging from superficial mucosal colonisation to life-threatening disseminated infections, particularly in critically ill, elderly, or immunocompromised patients (3). The increasing incidence of *C. glabrata* infections has been linked to the widespread use of broad-spectrum antifungal agents and invasive medical procedures, both of which have contributed to a shift in the epidemiology of fungal pathogens in healthcare settings (4).

Unlike *C. albicans*, *C. glabrata* is a haploid yeast phylogenetically closer to *Saccharomyces cerevisiae* and lacks the ability to form hyphae or pseudohyphae. Despite this, it compensates through a range of adaptive mechanisms that enhance survival and pathogenic potential within the host environment (5). These include remarkable metabolic flexibility, efficient nutrient scavenging, and robust tolerance to oxidative and nitrosative stresses generated by the host immune response.

Importantly, *C. glabrata* exhibits intrinsic low susceptibility to azole antifungals, primarily due to reduced intracellular drug accumulation and overexpression of efflux pumps. In addition, increasing rates of echinocandin resistance have been reported, frequently associated with mutations in the *FKS1* and *FKS2* genes, which encode catalytic subunits of β-1,3-D-glucan synthase (2, 6).

Virulence is further enhanced by the epithelial adhesin (EPA) gene family, which mediates adhesion to epithelial cells and abiotic surfaces such as indwelling medical devices, thereby promoting colonisation and biofilm formation (7, 8). Biofilms provide substantial protection against both host immune defences and antifungal agents, significantly complicating the management of device-associated infections (9).

Another important feature of *C. glabrata* pathogenicity is its ability to persist intracellularly within macrophages. The organism can survive within phagocytes by interfering with phagolysosomal maturation, modulating inflammatory responses, and resisting oxidative stress (10, 11). These adaptations may contribute to persistent or relapsing infections even in immunocompetent hosts (12). In addition, *C. glabrata* demonstrates marked tolerance to acidic environments and regulates intracellular pH through vacuolar transport systems and proteases, facilitating survival within hostile host niches (11, 13).

Clinically, infections caused by *C. glabrata* are associated with higher mortality and more frequent treatment failure compared with those caused by *C. albicans*. These outcomes are influenced not only by antifungal resistance but also by pharmacokinetic limitations of antifungal agents in poorly vascularised compartments such as abscesses, pleural cavities, and necrotic tissues (14).

Recent studies have described *C. glabrata* as a highly adaptable pathogen characterised by exceptional stress tolerance and the capacity to persist in challenging host environments (15). These features highlight the need for improved therapeutic strategies, including the potential role of combination antifungal therapy in selected cases where pharmacokinetic barriers or high fungal burden may limit the effectiveness of monotherapy.

Here, we present a clinically complex case of multidistrict *Candida glabrata* infection successfully managed with echinocandin–liposomal amphotericin B combination therapy, illustrating the therapeutic challenges posed by infections involving poorly perfused anatomical compartments.

## 2 Methods

### 2.1 Clinical management and sample collection

Following emergency surgical repair of the gastric perforation and diaphragmatic defect, the patient was admitted to the intensive care unit (ICU) for advanced haemodynamic and respiratory support. Management included invasive monitoring, mechanical ventilation, and broad-spectrum antimicrobial therapy according to ICU protocols.

Serial clinical specimens were collected throughout the postoperative period to evaluate fungal burden and monitor response to treatment. These included blood cultures, pleural aspirates, bronchoalveolar lavage (BAL) samples, surgical wound swabs, and urine specimens. Follow-up cultures were obtained at clinically relevant intervals based on the patient’s condition and microbiological monitoring protocols rather than through daily routine sampling.

All samples were obtained using strict aseptic techniques and processed according to international best-practice guidelines for the diagnosis and management of invasive candidiasis (3, 16).

### 2.2 Microbiological identification and antifungal susceptibility testing

All specimens were cultured on Sabouraud dextrose agar and CHROMID® Candida agar (bioMérieux, Marcy-l’Étoile, France) and incubated at 35 °C for 48 h. Colony morphology and pigmentation were recorded.

Species identification was confirmed using matrix-assisted laser desorption/ionisation time-of-flight mass spectrometry (MALDI-TOF MS; VITEK® MS, bioMérieux, Marcy-l’Étoile, France), yielding confidence scores ≥99%. The use of both culture-based and proteomic identification methods ensured accurate detection of *Candida glabrata* isolates across multiple anatomical sites.

Antifungal susceptibility testing was performed using the Sensititre™ YeastOne™ YO10 broth microdilution system (Thermo Fisher Scientific, USA) according to Clinical and Laboratory Standards Institute (CLSI) M27-A3 guidelines (16). The antifungal panel included amphotericin B, anidulafungin, caspofungin, micafungin, fluconazole, itraconazole, posaconazole, and voriconazole. Minimum inhibitory concentrations (MICs) were interpreted in accordance with CLSI clinical breakpoints and epidemiological cutoff values (2, 6). Quality control strains recommended by CLSI were used for antifungal susceptibility testing. These susceptibility results guided therapeutic decision-making and supported subsequent escalation of antifungal therapy. Direct microscopic examination (Gram staining) was performed on selected specimens, including pleural and peritoneal fluids, as part of routine microbiological processing.

### 2.3 Imaging and interventional procedures

Serial computed tomography (CT) scans of the chest and abdomen were performed to evaluate disease progression and monitor the response to treatment. Follow-up chest radiographs were also obtained during the ICU stay to assess pleural collections and pulmonary status.

Video-assisted thoracoscopic surgery (VATS) was performed for pleural drainage and repositioning of chest tubes when clinically indicated. Ultrasound guidance was used for interventional procedures and sampling to ensure procedural safety and accuracy.

### 2.4 Antifungal therapy protocol

Initial antifungal therapy consisted of caspofungin administered as a 75 mg loading dose followed by 50 mg daily, in accordance with current clinical guidelines for invasive candidiasis.

Due to persistent microbiological positivity and ongoing clinical instability, antifungal therapy was escalated to micafungin (100 mg daily) on postoperative day (POD) 16.

After 72 h of micafungin monotherapy without clear clinical or microbiological improvement, liposomal amphotericin B (450 mg/day) was added to the regimen. The decision to initiate combination therapy was based on pharmacokinetic considerations and supported by experimental evidence demonstrating potential synergistic activity between echinocandins and polyenes (17–19).

Combination therapy was continued until microbiological clearance was achieved, followed by progressive clinical stabilisation and complete recovery.

## 3 Results

### 3.1 Case presentation

A 39-year-old male, a chronic heavy smoker with no known immunosuppressive conditions, was admitted to the emergency department with septic shock secondary to gastric perforation into the thoracic cavity caused by a previously undiagnosed congenital diaphragmatic hernia. His past medical history included recurrent gastritis and surgical repair of a hiatal hernia during childhood, but no chronic systemic diseases, alcohol abuse, or immunosuppressive therapy.

Upon arrival, the patient presented with profound circulatory collapse characterized by undetectable blood pressure, tachycardia, and hypoxaemia despite supplemental oxygen. Clinical examination revealed a severe systemic inflammatory response with high-grade fever, altered mental status, and respiratory distress (respiratory rate 20–22 breaths/min) with bilaterally diminished breath sounds.

Laboratory investigations demonstrated marked leukocytosis, mild transaminitis (ALT 48 U/L, AST 55 U/L), hypoalbuminaemia (2.8 g/dL), metabolic acidosis with an increased anion gap, and elevated inflammatory markers. Arterial blood gas analysis confirmed compensated metabolic acidosis. Chest imaging revealed bilateral pneumothoraces (pleural air gap >1 cm) and a moderate right-sided pleural effusion.

Emergency surgery was performed, including partial gastrectomy and repair of the diaphragmatic defect. Due to extensive thoracic contamination from gastric contents, bilateral pleural drains were inserted. Following surgery, the patient was transferred to the intensive care unit (ICU), where he required mechanical ventilation and high-dose vasoactive support.

Computed tomography (CT) imaging revealed bilateral pleural fluid collections and intra-abdominal free fluid. Empirical antimicrobial therapy was initiated with meropenem, teicoplanin, clindamycin, and caspofungin (75 mg loading dose followed by 50 mg daily). On postoperative day (POD) 5, persistent pneumothorax and pleural effusion required thoracoscopic revision and repositioning of chest drains.

Despite these interventions, serial blood and pleural fluid cultures obtained between POD 8 and POD 16 yielded yeast-like colonies subsequently identified as *Candida glabrata* using MALDI-TOF MS (VITEK-MS, bioMérieux) after primary growth on CHROMID® Candida agar. Antifungal susceptibility testing demonstrated reduced susceptibility to azoles but preserved susceptibility to echinocandins and amphotericin B.

The patient remained critically ill with persistent fever, haemodynamic instability, and continued microbiological positivity from multiple sites including blood, pleural aspirates, surgical wound swabs, and bronchoalveolar lavage (BAL) samples. Detection of *Candida* species in BAL samples was interpreted cautiously, as respiratory specimens may reflect colonisation rather than true invasive infection; however, the presence of concomitant candidemia and pleural infection supported the interpretation of a multidistrict infectious process.

On POD 16, antifungal therapy was escalated to micafungin (100 mg/day). After 72 h without clear clinical or microbiological improvement, liposomal amphotericin B (450 mg/day) was added to enhance tissue penetration and exploit potential synergistic antifungal effects.

Following initiation of combination therapy, the patient demonstrated rapid clinical improvement. Fever resolved within 96 h, haemodynamic stability was restored, and microbiological clearance was achieved across all sampled sites by POD 20. The patient was subsequently weaned from mechanical ventilation and vasoactive support, and pleural drains were removed. He was discharged on POD 30 in stable condition, afebrile and without evidence of ongoing infection (see Table 1).

**Table 1:** Temporal evolution of inflammatory markers and clinical management.

Time point	CRP (mg/L)	Procalcitonin (ng/mL)	WBC (×10^9^/L)	Clinical management
Admission	178	12.44	3.74	Septic shock; norepinephrine infusion; caspofungin loading dose (70 mg)
Day 5	114	3.20	5.99	Norepinephrine discontinued; switch to micafungin (100 mg/day)
Day 7	169	0.75	13.37	Persistent infection despite antifungal therapy
Day 11	129	0.60	11.51	Ongoing antifungal treatment
Day 13	120	0.35	10.35	Micafungin therapy continued
Day 16	58	0.22	9.28	Liposomal amphotericin B added to micafungin
Day 18	42	0.13	7.30	Clinical improvement and decline of inflammatory markers
**Day 20**	**25.7**	—	**5.42**	**Microbiological clearance achieved**
**Day 28**	**26.5**	**0.10**	**6.38**	Follow-up evaluation; persistent clinical stability and microbiological negativity

Serial measurements of C-reactive protein (CRP), procalcitonin (PCT), and white blood cell count (WBC) are shown in relation to major therapeutic interventions. A progressive decline in inflammatory markers was observed following escalation to combination antifungal therapy with micafungin and liposomal amphotericin B, coinciding with microbiological clearance. Follow-up laboratory evaluation at day 28 confirmed sustained clinical improvement. Bold values indicate key clinical turning points corresponding to major therapeutic changes and microbiological clearance.

### 3.2 Microbiological findings

*Candida glabrata* was repeatedly isolated from multiple anatomical sites, including blood cultures, pleural aspirates, surgical wound swabs, bronchoalveolar lavage (BAL) samples, and urine specimens. Identification by MALDI-TOF MS consistently yielded high-confidence scores (≥99%), confirming the presence of the same species across all compartments.

Antifungal susceptibility testing revealed markedly reduced susceptibility to azoles, with fluconazole MIC >32 μg/mL and voriconazole MIC >4 μg/mL. In contrast, susceptibility to echinocandins and amphotericin B was retained (caspofungin MIC 0.06 μg/mL; micafungin MIC 0.03 μg/mL; amphotericin B MIC 0.5 μg/mL). The amphotericin B MIC value was below the CLSI epidemiological cutoff value (2 mg/L).

All isolates exhibited identical antifungal susceptibility profiles, supporting the presence of a single infecting strain across the different anatomical sites.

Persistent fungal growth was documented between POD 8 and POD 16 during echinocandin-based therapy. Following initiation of combination therapy with micafungin and liposomal amphotericin B, cultures obtained on POD 20 from blood, BAL, and urine samples became negative, and no regrowth was observed in subsequent surveillance cultures. No bacterial pathogens were isolated from blood, pleural fluid, bronchoalveolar lavage, or other clinical specimens during the course of hospitalization (see Table 2).

**Table 2:** Antifungal susceptibility profile of *Candida glabrata* isolates recovered from blood, pleural fluid, and peritoneal samples.

Antifungal	Blood isolate MIC	Peritoneal isolate MIC	Pleural isolate MIC
Caspofungin	0.06	0.06	0.06
Micafungin	0.015	0.015	0.015
Anidulafungin	0.03	0.03	0.03
Amphotericin B	–	1	–
Fluconazole	–	8	–
Voriconazole	–	0.5	–

Minimum inhibitory concentrations (MICs) were determined according to CLSI criteria. The isolates showed preserved susceptibility to echinocandins and amphotericin B, whereas reduced susceptibility or resistance to azoles was observed. This susceptibility pattern supported escalation to combination therapy with micafungin and liposomal amphotericin B in the context of persistent multidistrict infection.

### 3.3 Clinical course and therapeutic response

Despite early echinocandin therapy, the patient remained febrile (temperature >38.5 °C) and haemodynamically unstable, with persistent microbiological positivity for *C. glabrata*. Radiological follow-up demonstrated persistent pleural collections and localized fluid accumulations consistent with ongoing infection.

Although partial microbiological control during caspofungin therapy may have contributed to initial bloodstream infection management together with surgical source control, persistent positivity in other anatomical compartments suggested incomplete eradication of the pathogen.

Escalation to micafungin monotherapy (100 mg/day) did not result in significant clinical improvement. However, within 96 h of initiating liposomal amphotericin B in combination with micafungin, the patient demonstrated marked clinical recovery. Fever subsided, inflammatory markers declined, and haemodynamic parameters stabilised without the need for further vasoactive support.

Repeat imaging showed near-complete resolution of pleural collections, allowing removal of chest drains. By POD 22 the patient was successfully weaned from mechanical ventilation and transferred from the ICU to a general ward.

He was discharged on POD 30 in stable clinical condition with all microbiological cultures remaining negative. Follow-up evaluation 4 weeks after discharge showed no evidence of relapse or residual infection.

**Figure 1:**
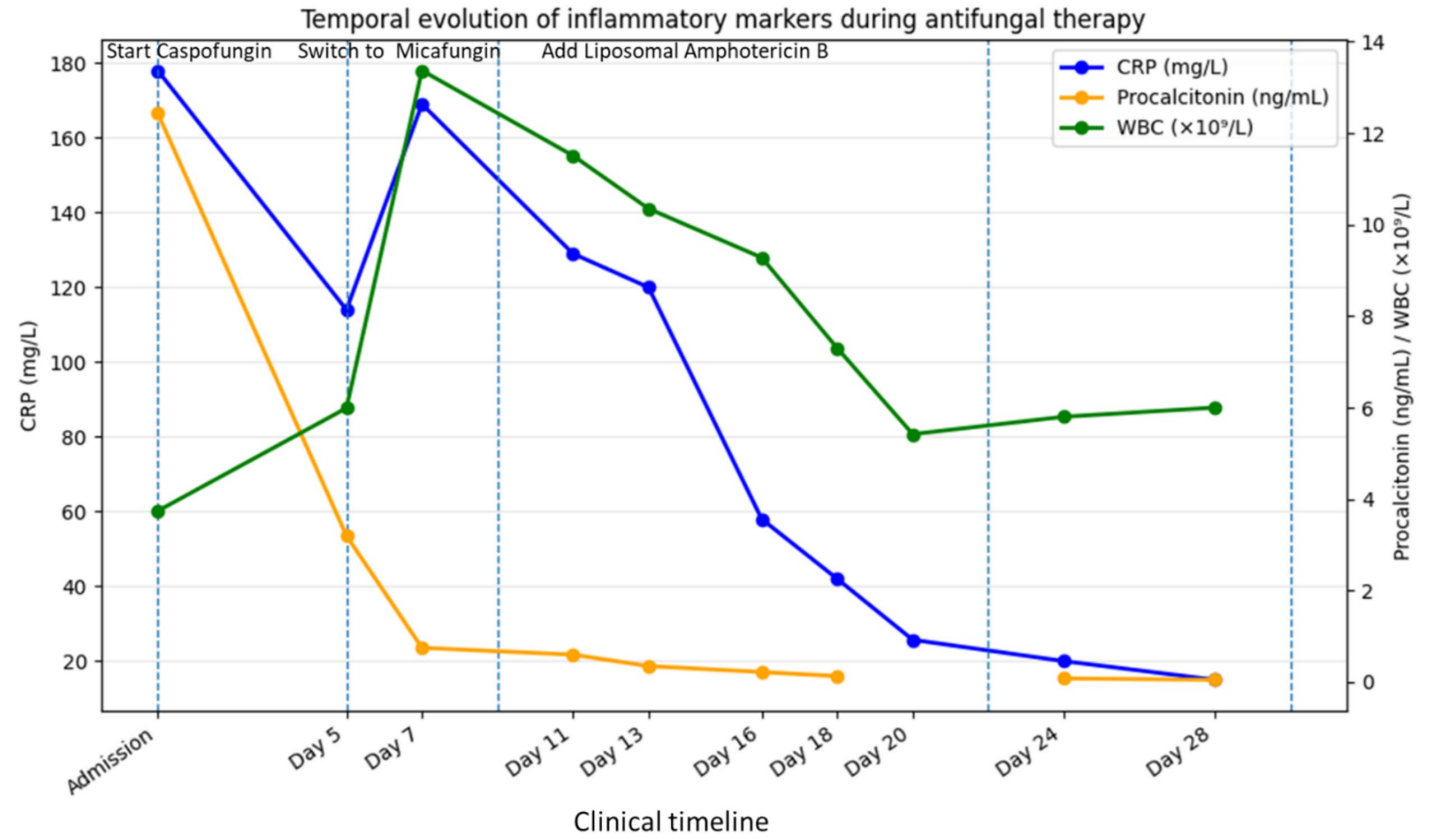
Temporal evolution of inflammatory markers during antifungal therapy. Changes in C-reactive protein (CRP), procalcitonin (PCT), and white blood cell count (WBC) during hospitalization are shown in relation to key therapeutic interventions. Initial treatment with caspofungin was followed by escalation to micafungin and subsequent addition of liposomal amphotericin B. The progressive decline of inflammatory markers parallels microbiological clearance and clinical recovery.

The temporal evolution of inflammatory markers during hospitalization is shown in Figure 1. A progressive decline in CRP and procalcitonin was observed following escalation to combination antifungal therapy (see Table 3).

**Table 3:** Temporal evolution of microbiological findings during hospitalization.

Date	Clinical phase	Sample site	Microbiological result
08	Early postoperative phase	Blood culture	*Candida glabrata* detected
09	Early postoperative phase	Blood culture	*Candida glabrata* detected
10	Early postoperative phase	Blood culture	*Candida glabrata* detected
11	Early postoperative phase	Surgical wound	*Candida glabrata* detected
12	Early postoperative phase	Pleural fluid	*Candida glabrata* detected
16	Persistent infection phase	BAL, wound swab	*Candida glabrata* detected
20	After combination therapy	Blood, BAL, urine	Negative
21	Microbiological clearance	All sampled sites	Negative
28	Follow-up evaluation	All sampled sites	Negative

*Candida glabrata* was repeatedly isolated from multiple anatomical compartments including blood, pleural fluid, bronchoalveolar lavage (BAL), and surgical wound samples during the early postoperative period. Microbiological clearance was achieved after escalation to combination antifungal therapy with micafungin and liposomal amphotericin B. Follow-up cultures performed confirmed persistent microbiological negativity.

## 4 Discussion

Invasive candidiasis remains a major cause of morbidity and mortality among critically ill patients, with *Candida glabrata* representing a clinically challenging pathogen due to its antifungal resistance profile and persistence in host environments.

The clinical behavior of *C. glabrata* is largely determined by its unique biological characteristics. Although this species lacks the ability to form hyphae, it compensates through several adaptive mechanisms that enhance survival within the host environment. These include strong adhesion mediated by epithelial adhesins (EPA family), efficient metabolic adaptation to nutrient-limited conditions, and the ability to persist intracellularly within macrophages (5, 7–12). In addition, robust stress-response pathways allow the organism to tolerate oxidative stress and acidic environments encountered within host tissues (11, 13).

These biological features contribute to the ability of *C. glabrata* to persist in multiple anatomical compartments and may explain the development of multifocal infections even in immunocompetent individuals. In the present case, septic shock may also have contributed to transient immune dysregulation, potentially impairing macrophage and neutrophil function and thereby facilitating intracellular persistence of the pathogen despite antifungal therapy.

The present report illustrates how *C. glabrata* infection can simultaneously involve several anatomical sites—including the bloodstream, pleural space, surgical wound, and respiratory tract—creating a complex therapeutic scenario. Multidistrict involvement may significantly complicate management, particularly when infected compartments are poorly vascularised.

**Figure 2:**
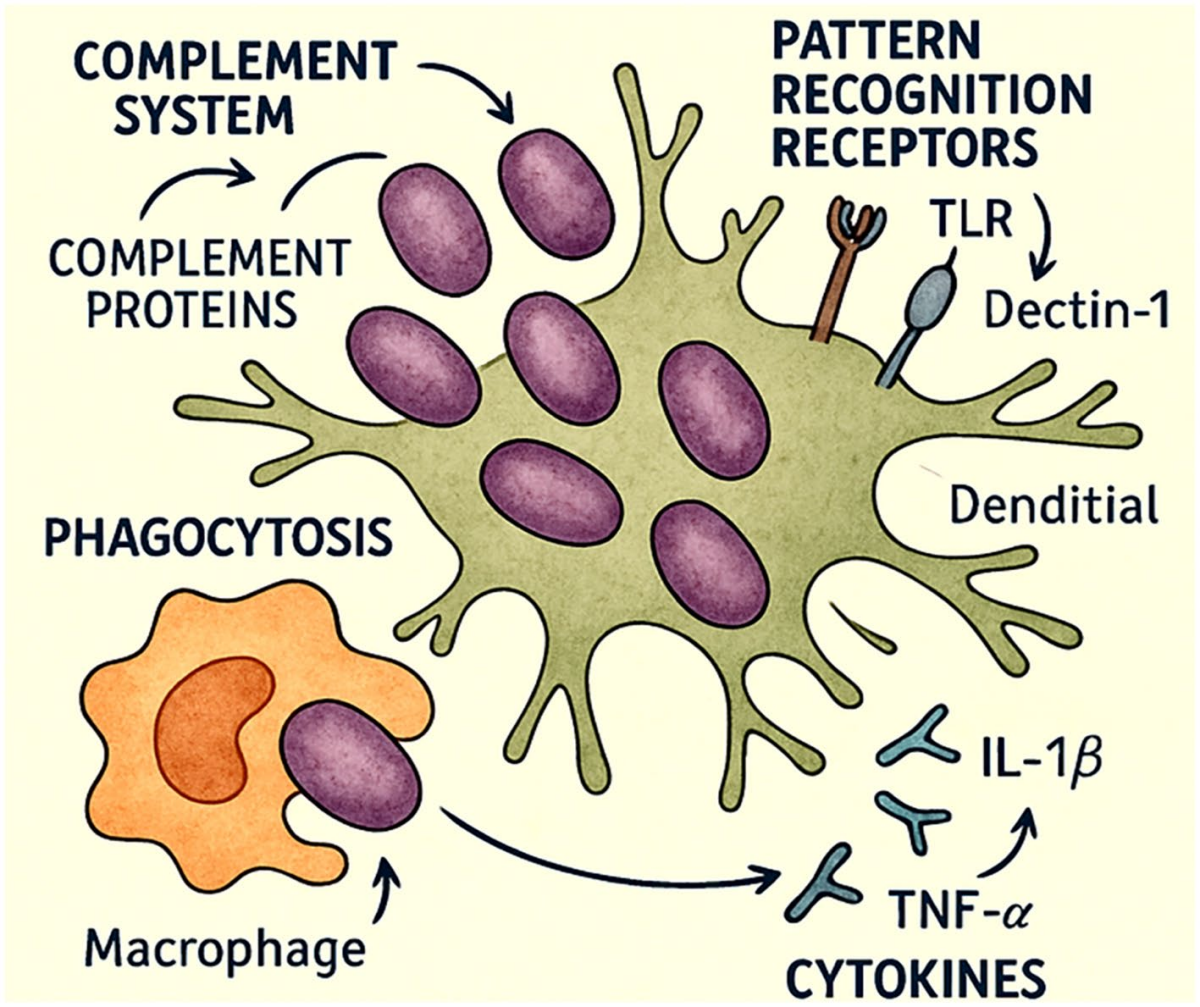
Immunological features of *C. glabrata*. The complement system opsonises fungal cells and promotes lysis. Pattern-recognition receptors—such as toll-like receptors (TLRs) and Dectin-1—detect fungal antigens and trigger innate immune signalling. Macrophages phagocytose invading microorganisms, while pro-inflammatory cytokines including interleukin-1β (IL-1β) and tumour necrosis factor-α (TNF-α) amplify the inflammatory response. Dendritic cells bridge innate and adaptive immunity by presenting fungal antigens and activating antigen-specific T-cell responses.

Among these sites, the pleural cavity represents a particularly challenging therapeutic environment. Limited vascularisation, the frequent presence of loculated collections, and restricted antifungal penetration may reduce the effectiveness of systemic therapy (17, 20). Consequently, even when isolates appear susceptible *in vitro*, pharmacokinetic constraints may result in suboptimal antifungal exposure at the site of infection (see Figure 2).

Another important challenge in the management of *C. glabrata* infections is its antifungal resistance profile. This species exhibits intrinsic reduced susceptibility to azole antifungals, largely due to efflux pump overexpression and alterations in ergosterol biosynthesis pathways (4, 6). In addition, echinocandin resistance may emerge through mutations in the *FKS1* and *FKS2* genes encoding β-1,3-D-glucan synthase (2, 6, 21). Nevertheless, treatment failure may occur even in isolates that remain phenotypically susceptible. In such cases, pharmacokinetic factors and the anatomical location of infection may play a major role. Both caspofungin and micafungin achieve high plasma concentrations but exhibit limited penetration into certain compartments, including pleural fluid and abscess cavities (16, 19, 22).

In the present patient, partial microbiological control during initial caspofungin therapy may have contributed to bloodstream infection management in combination with surgical source control. However, persistent fungal positivity in other compartments suggested incomplete eradication of the pathogen. The combination of high fungal burden, multifocal infection, and limited drug penetration into pleural collections likely contributed to the persistence of infection despite persistence of infection despite partial microbiological control achieved during echinocandin therapy.

In recent years, combination antifungal therapy has gained increasing attention as a potential strategy in selected cases of invasive candidiasis characterised by high fungal burden, pharmacokinetic barriers, or emerging resistance (18, 23–25). The rationale for this approach lies in the complementary pharmacodynamic mechanisms of the drugs involved.

**Figure 3:**
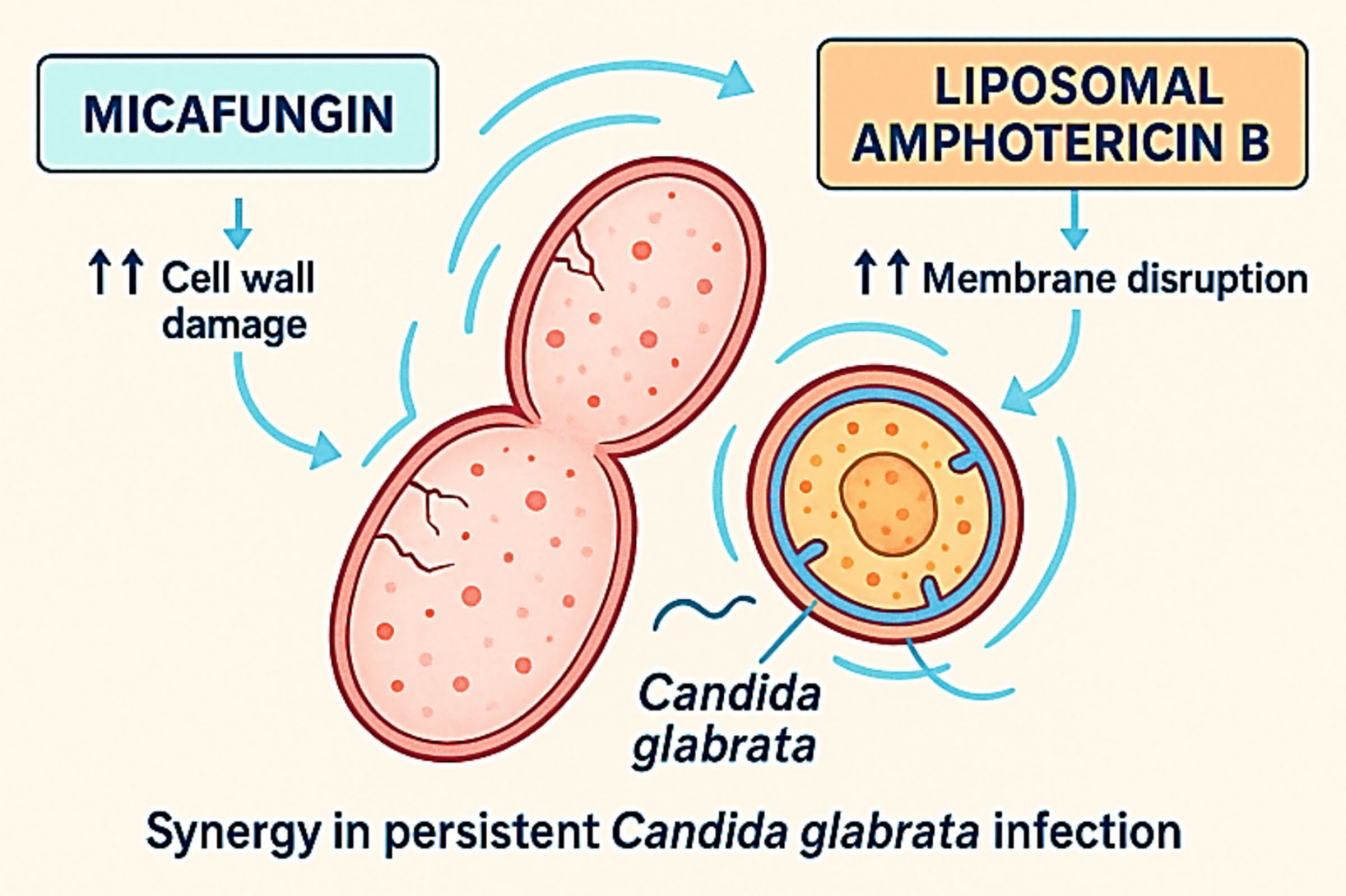
Synergistic antifungal mechanisms of micafungin and liposomal amphotericin B against *C. glabrata*. Micafungin inhibits β-1,3-D-glucan synthase, causing progressive cell wall damage and structural weakening. Liposomal amphotericin B binds to membrane ergosterol, inducing pore formation and membrane disruption. The combination results in complementary cell wall and membrane injury, enhancing fungicidal activity and promoting rapid clearance in persistent *C. glabrata* infection.

Echinocandins inhibit the β-1,3-D-glucan synthase complex, weakening the fungal cell wall and increasing osmotic fragility (6, 23). Amphotericin B, by contrast, binds to ergosterol in the fungal cell membrane, creating pores that disrupt membrane integrity and lead to rapid cell death (17, 19). When administered together, these mechanisms may act synergistically: cell wall disruption induced by echinocandins can facilitate deeper amphotericin B penetration, while membrane destabilisation amplifies the stress imposed on fungal cells. Experimental studies have described this interaction as a “one-two punch” antifungal effect, resulting in enhanced fungicidal activity and delayed resistance emergence (18, 24, 26) (see Figure 3).

Pharmacokinetically, the two drugs complement each other. Echinocandins achieve high plasma concentrations and prolonged exposure in well-perfused organs such as the liver and bloodstream but have poor distribution in poorly vascularised compartments such as pleural spaces or abscesses (16, 19, 22). Liposomal amphotericin B, by contrast, exhibits superior tissue penetration, excellent distribution into complex anatomical niches—including empyematous and necrotic cavities—and an extended half-life that sustains therapeutic levels even in pharmacological “sanctuary sites” (14, 17, 19). Furthermore, liposomal formulations mitigate amphotericin-related nephrotoxicity and enhance macrophage uptake, improving delivery to intracellular and biofilm-associated fungal populations (17, 19, 25). This dual-target, pharmacokinetically optimised approach is particularly advantageous in multicompartment infections—such as those affecting the bloodstream, pleural space, and surgical sites—where monotherapy may fail due to incomplete drug penetration or sublethal exposure. In our case, the combination of micafungin and liposomal amphotericin B produced rapid defervescence, microbiological clearance, and complete clinical recovery. Adequate surgical source control also likely contributed to infection resolution in this case. These results align with accumulating clinical and experimental evidence indicating that echinocandin–polyene combinations can outperform monotherapy in refractory or multidistrict *Candida* infections by enhancing killing efficiency and mitigating the development of resistance (18, 23–27) (see Table 4).

**Table 4:** Comparative pharmacologic characteristics and clinical implications.

Parameter	Echinocandins	Liposomal amphotericin B
Primary mechanism of action	Inhibition of β-1,3-D-glucan synthase → loss of cell-wall integrity	Binds membrane ergosterol → pore formation and membrane depolarisation
Fungicidal activity	Fungicidal against Candida spp., including *C. glabrata*, although activity may be influenced by infection burden and anatomical compartment	Broadly fungicidal against *Candida* spp., including resistant strains
Pharmacokinetics (PK)	High plasma levels, low Vd; poor penetration into pleural, peritoneal, and CNS compartments	Broad tissue distribution; excellent penetration into pleural, peritoneal, and abscess compartments
Half-life/elimination	10–15 h (micafungin); predominantly hepatic metabolism	Biphasic: initial 5–10 h; terminal ≈100–150 h due to tissue sequestration and slow redistribution
Protein binding	>95%; concentration-dependent	≈90%; extensive tissue affinity mediated by lipid carrier
Renal toxicity	Minimal	Reduced compared to conventional amphotericin B, but still higher than echinocandins
Clinical strengths	Excellent systemic coverage for candidemia and early fungistatic control	Superior deep-tissue sterilisation; efficacy in refractory, multidistrict, or biofilm-associated infections
Limitations	Limited penetration into ‘pharmacologic sanctuaries’; emerging *FKS1/FKS2* resistance	Potential nephrotoxicity; infusion-related reactions; higher cost
Synergistic potential	Weakens cell wall, facilitating AmB penetration	Membrane disruption potentiates echinocandin-induced stress → “one-two punch” synergistic fungicidal effect
Clinical application in this case	Initial monotherapy (insufficient in multidistrict infection)	Added to achieve pleural and surgical-site sterilisation; rapid clearance and defervescence
Overall therapeutic role	Provides rapid systemic coverage and early fungistatic control	Ensures deep-compartment fungicidal sterilisation and resistance suppression

Comparative pharmacologic characteristics and clinical implications of echinocandins and liposomal amphotericin B relative to invasive *C. glabrata* infections. Their complementary pharmacokinetic and pharmacodynamic properties justify combined use in refractory or compartmentalised disease. Echinocandins provide rapid systemic control, whereas liposomal amphotericin B ensures deep-tissue sterilisation and improved eradication of fungal reservoirs.

## 5 Conclusion

Invasive infections caused by *Candida glabrata* represent a significant clinical challenge because of the organism’s intrinsic antifungal resistance profile, adaptive virulence mechanisms, and ability to persist in hostile host environments. Management may be particularly complex when infections involve multiple anatomical compartments or poorly vascularised sites, where antifungal penetration can be limited.

The present case illustrates the potential difficulties encountered when treating multidistrict *C. glabrata* infections, especially in the context of critical illness and complex surgical complications. Although echinocandins remain the recommended first-line therapy for invasive candidiasis, pharmacokinetic constraints and compartmentalised infection may limit the effectiveness of antifungal monotherapy in certain clinical scenarios.

In this patient, escalation to combination therapy with micafungin and liposomal amphotericin B was associated with rapid clinical improvement and microbiological clearance. The complementary pharmacodynamic mechanisms and tissue distribution profiles of these agents may provide advantages in infections involving poorly perfused anatomical compartments.

Early recognition of persistent fungal infection, appropriate source control, and careful consideration of pharmacokinetic and pharmacodynamic factors may help optimise treatment strategies in complex cases of invasive candidiasis.

However, this report describes a single clinical case, and causal relationships between combination antifungal therapy and the favourable outcome cannot be definitively established. To our knowledge, reports describing multidistrict Candida glabrata infections successfully treated with echinocandin–liposomal amphotericin B combination therapy remain extremely limited. Further prospective studies and controlled clinical investigations are needed to clarify the role, safety, and optimal timing of echinocandin–amphotericin B combination therapy in the management of invasive *Candida glabrata* infections (28–30).

## 6 Patient perspective

After discharge the patient reported gradual return to normal daily activities and expressed gratitude for the multidisciplinary care received during hospitalisation. At four-week follow-up he remained asymptomatic and had resumed normal physical activity.

## Data Availability

The raw data supporting the conclusions of this article will be made available by the authors, without undue reservation.
